# Zinner Syndrome: A Case Report of a Rare Etiology of Infertility

**DOI:** 10.7759/cureus.17386

**Published:** 2021-08-23

**Authors:** Abdulrhman M Almofareh, Khalid M Aljuaid, Sewar A Almirabi, Yazn M Hakami, Amin M Alwan

**Affiliations:** 1 Urology, Batterjee Medical College, Jeddah, SAU; 2 Urology, Ibn Sina National College for Medical Studies, Jeddah, SAU

**Keywords:** unilateral renal agenesis, male factor infertility, seminal vesicle cyst, zinner syndrome, case report

## Abstract

Infertility is a common medical issue with different etiologies. It can be related to female factors, male factors, or factors related to both partners. We report the case of a 35-year-old male patient who presented with primary infertility for 10 years. He was otherwise healthy with unremarkable past medical history. Physical examination revealed normal external genitalia with both testes were normal in size. Basic semen analysis revealed decreased sperm volume and sperm count. Abdominal ultrasound examination revealed an absent right kidney. Subsequently, the patient under a computed tomography scan that confirmed the right renal agenesis and demonstrated a well-defined right seminal vesicle cyst. Such findings were consistent with the diagnosis of Zinner syndrome. He underwent aspiration of the cyst that resulted in improvement in the sperm parameter of basic semen analysis. The case demonstrated a rare etiology of male infertility that was successfully managed conservatively. Despite its rarity, physicians should consider the developmental anomalies of the genitourinary system when encountering patients with infertility.

## Introduction

Infertility is a prevalent health condition affecting up to 10% of the population and has significant psychological, economic, and medical implications [1]. It is defined as the inability of a couple to conceive after 12 months despite regular sexual intercourse without the use of contraceptives [1]. Epidemiological studies revealed that up to 90% of couples usually conceive within the first year of attempted conception [2]. Infertility is a unique condition as it could be related to female factors, male factors, or factors contributed by both partners. Male infertility could be due to several etiologies, including endocrine disorders, primary testicular defects in spermatogenesis, sperm transfer disorders, or idiopathic male infertility. Herein, we present the case of a middle-aged man with primary infertility who was found to have a rare condition, known as Zinner syndrome, which is defined as the presence of a congenital seminal vesicle cyst.

## Case presentation

We report the case of a 35-year-old male patient who presented to the infertility clinic with primary infertility for 10 years. He has a regular sexual activity every three days with no difficulties related to libido or potency. He had no urinary complaints. He has no history of smoking or alcohol drinking. The family history was unremarkable for infertility. The past medical history was non-contributory.

His spouse is a 30-year-old woman with no health issues. She has regular menstrual cycles with no history of dysmenorrhea or menorrhagia. She had not undergone any operations in the past and had no history of sexually transmitted diseases. They reported that the problem of infertility was having a significant psychological impact on their lives.

Physical examination of the patient showed normal abdominal and genital examination with both testes were in size. Physical examination of the spouse revealed a soft abdomen with no masses or tenderness. Bimanual pelvic examination was normal.

Basic semen analysis was performed for the patient, which revealed a normal semen color and viscosity, a semen volume of 2 ml, and a sperm count of 10 million. Basic laboratory investigations, including hematological and biochemical tests, were within normal limits. Otherwise, his spouse had a normal hormonal profile with an unremarkable ultrasonic examination of the pelvis. Additionally, her hysterosalpingography examination showed a normal uterus and patent fallopian tubes.

**Figure 1:**
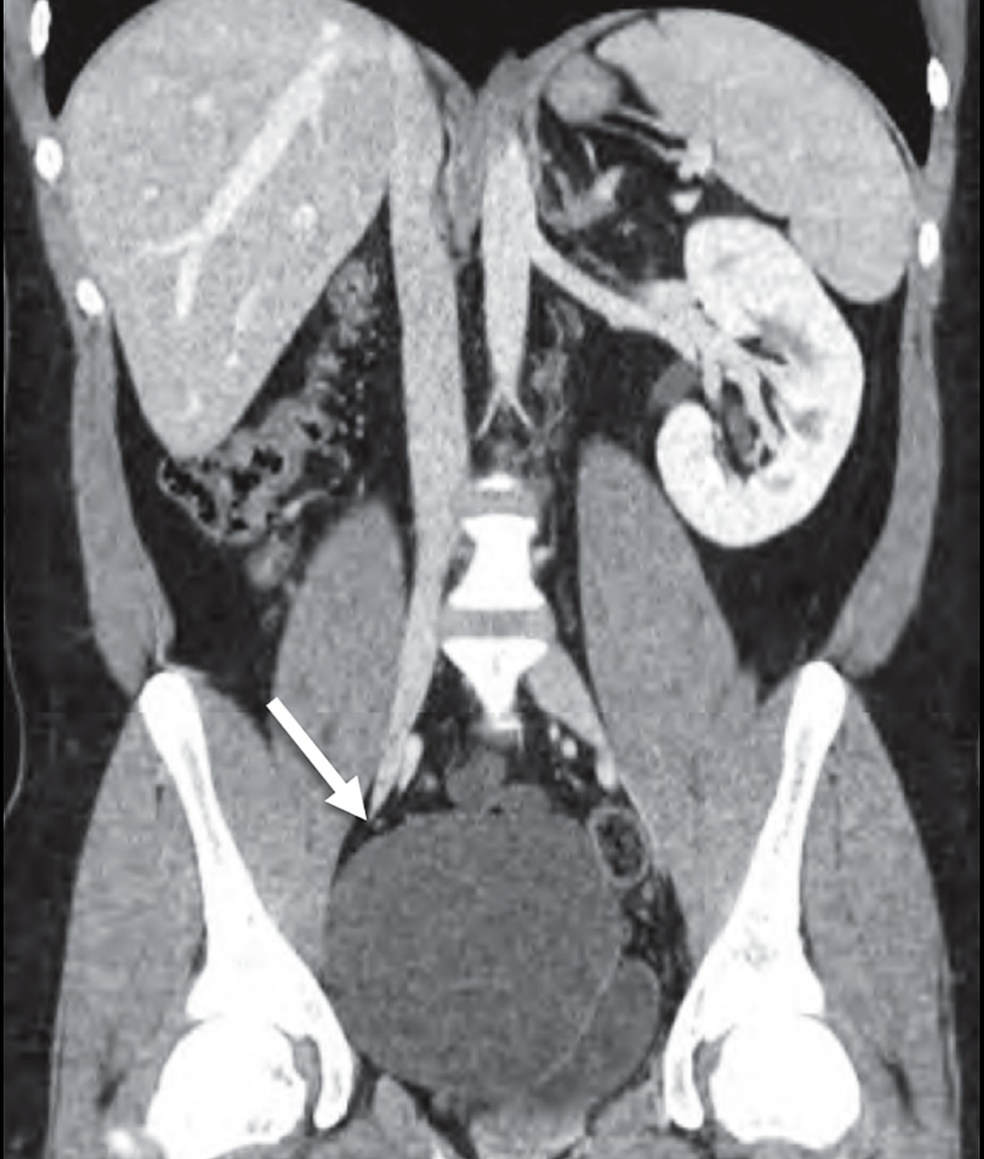
Coronal computed tomography image. Coronal computed tomography image of the abdomen showing a right-sided well-defined cyst in the pelvis (arrow) with an absent right kidney.

**Figure 2:**
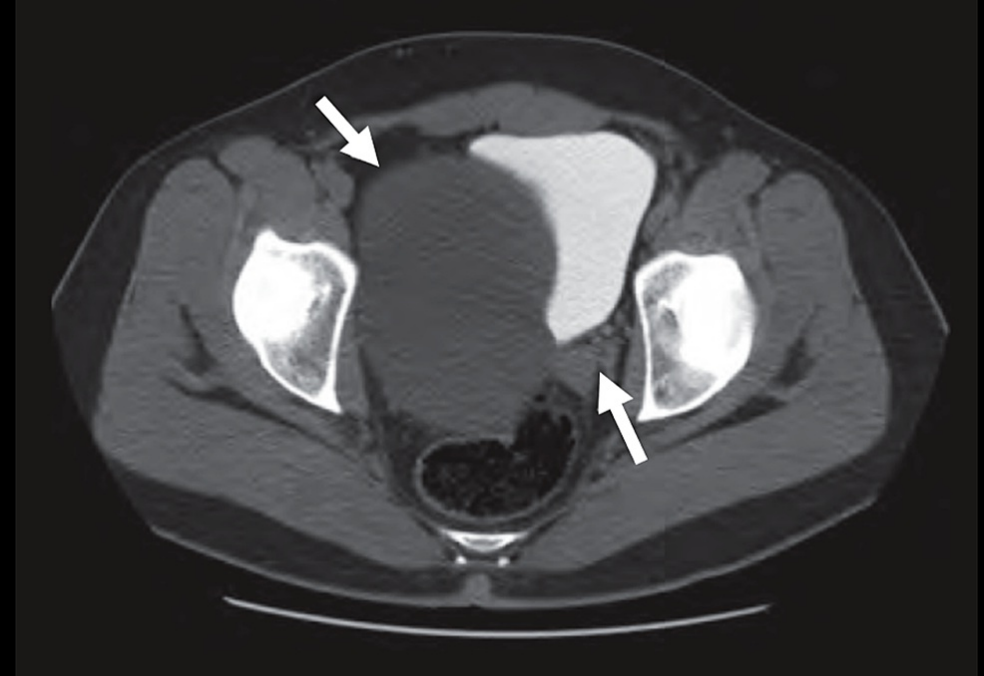
Axial computed tomography image. Axial contrast-enhanced computed tomography image of the pelvis (excretory phase) showing a well-defined cyst in the pelvis displacing the urinary bladder to the left (arrows).

Subsequently, the patient underwent a bedside ultrasound which revealed an absence of the right kidney. Computed tomography examination confirmed the finding of right renal agenesis and a well-circumscribed cyst in the pelvis (Figure 1, 2). Such findings were consistent with the diagnosis of Zinner syndrome.

Accordingly, the patient was planned to have a transurethral aspiration of the seminal vesicle cyst combined with minocycline sclerotherapy. Analysis of the aspirated fluid revealed spermatozoa. A few months later, the patient repeated the basic semen analysis and showed improvement in the sperm parameters. 

## Discussion

We reported a case of Zinner syndrome, which is a rare congenital genitourinary anomaly that was first described in 1914 [3]. It is characterized by a trial of ejaculatory duct obstruction, seminal vesicle cyst, and ipsilateral agenesis. The clinical manifestation of Zenner syndrome includes dysuria, frequency, perineal pain, and infertility.

The common embryologic origin of the genital and renal systems explains the association of seminal vesicle cyst and the ipsilateral renal agenesis in this syndrome. It is thought to be related to an in-utero insult resulting in maldevelopment of the distal Wolffian duct. Hence, some authors describe this syndrome as the male counterpart of the Mayer-Rokitansky-Kuster-Hauser syndrome [4].

The diagnosis of Zinner syndrome can accurately be reached by imaging. Magnetic resonance imaging is the modality of choice to evaluate the anatomy of the genital tract. However, since it was not available in our institution, a computed tomography scan was performed.

The management of Zinner syndrome depends on the clinical manifestation. Regular surveillance is sufficient for asymptomatic patients. Aspiration of the seminal vesicle cyst can be curative as in the present case. Surgical treatment and assisted reproductive techniques, such as in-vitro fertilization can be offered if the conservative management was not successful [5]. Laparoscopic management of the cyst seems a feasible surgical option [6].

## Conclusions

The case demonstrated a rare etiology of male infertility that was successfully managed conservatively. Despite its rarity, physicians should consider the developmental anomalies of the genitourinary system when encountering patients with infertility. Imaging modalities have the ability to make an accurate diagnosis of such anomalies.
